# Ulceration and Calcareous Concretion at Aortic Valves—Dilatation of Mitral—Hypertrophy—Disease of Kidneys—Arachnitis

**Published:** 1842-09-03

**Authors:** 


### CLINICAL REPORTS.

### *Blockley Hospital—Service of W. W. Gerhard, M.D.*

Reported by J. L. LUDLOW, M. D., Resident Physician.

*Ulceration and Calcareous Concretion at Aortic Valves—Dilatation of Mitral—Hypertrophy—Disease of Kidneys—Arachnitis.*

Peter Halk, æt. 33, a German by birth, and by trade a cooper, states that he was healthy till five weeks since, when his abdomen enlarged very suddenly. His appetite is perhaps greater now than previously.

January 25. The patient is anemic; skin pale and bloodless, not jaundiced; eyes clear; countenance anxious; pulse slow, and regular, and full, 75; slight ascites, and œdema of the eyes and lips. *Heart.*—The first sound rough and sharp; second nearly lost in a bellows sound. The impulse of the heart is also increased, and the percussion dull. Respiration vesicular, except at the lower portion of the right lung, where there is a slight subcrepitant rhonchus. The percussion is also dull. Urine high coloured and abundant. Albuminous deposit by addition of nitric acid. Tenderness in the right hypochondrium.

℞. Ferri Proto-carb. (Valet,) gr. vj. Ter in die.
Senna et Magnes. Sulph. Nocte.

No notes taken until the

2d of March. Yesterday the patient was taken with œdema of the face and eyelids; to-day erysipelatous redness of the left eye; œdema of the legs; febrile excitement increased; pulse more excited; bellows sound of heart more developed; has some diarrhœa.

℞. Mist. Oleag.
C. Cups to heart, and iron stopped.

3. Œdema of the face not as great as yesterday, and the erysipelatous blush gradually disappearing from the eye; œdema of legs as yesterday; febrile excitement not so great; pulse 88; bowels not open during the night, though there was considerable tenesmus. Mist. Oleag. stopped, and

℞. Ol. Ricini, ℥j.

4. Pulse 116, frequent and thrilling; dyspnœa; bowels open twice during night; tongue has returned to its natural condition; anorexia almost complete. Heart—Bellows sound sharper than before, (churning or sawing motion with a creaking sound heard now and then,) extending to arch of aorta. Both sounds confused. Respiration feeble at the posterior inferior portion of the right lung, in consequence of effusion. Some tympanitis; more œdema in legs. The patient has had a peculiar form of fever, which has existed for some days, probably owing to aortitis.

℞. Emp. Episp. over heart.

5. Complains of no pain; pulse 80, not as thrilling as yesterday; skin moist; respiration more easy, though there is still slight dyspnœa. Countenance improved in appearance. Bowels open four times during the night, but no tenesmus.

Treatment continued.

6. Patient as yesterday. Pulse 120, full and resisting; respiration 22, high and laboured, with dilatation of the nostrils; bowels open three times last night, and once this morning; slept well during the day, but restless at night. Has pain over both kidneys.

℞. Dry Cups over kidneys, followed by sinapisms.

Pulse at night 108. Bowels open five times to-day.

℞. Dry Cups repeated, and pediluvium.

7. Oppression very great; pulse 116; skin moderately warm; respiration 40, feeble at the posterior lower portion of right lung; abdomen more tense; bowels open three or four times this morning. Action of the heart feeble; sounds not materially altered; flatness over the cardiac region more extended.

℞. Spts. Æth. Sulph. Comp. ʒj. Ter in die.

℞. Pulv. Doveri. Nocte.

In the evening found the patient much worse; tongue protruded to the left side, and unable to speak; immediately previous to this state he had complained of universal pain; no oppression; pulse quick and thrilling.

℞. Dry Cups to nucha and temples, and blister to nucha; sinapisms to the inside of the extremities; and Ol. Terebinth. enema.

8. Insensible; respiration stertorous.

Died to-day.

It may here be remarked, that the patient had not evinced even the ordinary brightness of intellect during the course of his sickness. But, on the contrary, answered the questions put to him in a single phrase. There was not, however, any marked cerebral symptoms evinced till the day of his death and the evening previous.

*Autopsy.*

Upon opening the skull, found the dura mater more adherent than natural, and ℥ss. of serum effused in the great cavity of the arachnoid. Upper surface of brain covered with yellow lymph-like exudation beneath; arachnoid at the anterior surface, one-twelfth of an inch in thickness, and dipping in between the convolutions. The posterior third has the arachnoid brightly injected, the yellow lymph disappearing. By moderate effort the arachnoid membrane may be detached from the surface of the brain. Lymph was also found moulded in the different depressions, on the anterior surface of the encephalic mass. Veins are only slightly injected. Where the surface of the brain was freed from lymph, the cortical portion was found firm, and only a little more pale than natural.

On the right hemisphere the appearances were similar to those on the left.

The ventricles contained about ℥ijss. of serum, of a turbid appearance. The membrane lining the ventricles was slightly opaque; the central portion was softened and diffluent. At the junction of the plexus choroides of the two sides a small body of medullary matter was found about the size of a filbert, much softened.

Meninges of the cerebellum at the anterior portion contain lymph similar to the cerebrum, in extent of surface about one and a half inches, and about two in breadth. The investing membrane of the medulla oblongata was injected. The fissures of Sylvius contain also small portions of lymph, similar to that on the upper surface of brain. The remainder of the brain was slightly injected throughout. The general mass of the brain was more flaccid and paler than usual, and an odour, of a peculiar alkaline character, was emitted on opening the cranium.

*Liver.*—Nutmeg coloured cyrrhosis, and fatty in colour and consistence.

*Lung.*—Slight cadaveric congestion; opaque at the upper surface.

*Kidneys*—Are one-half larger than natural; portions of both injected of a bright red colour, extending through the cortical substance, and then disposed so as to include the opaque yellow granules which replace it. The portion of the substance which is natural, contrasts strongly with the morbid appearances before mentioned.

On the external portion, the bloodvessels surround the acini in a very marked manner.

*Heart*—Is of twice the usual size; hypertrophy of both ventricles.

The pericardium contained about ℥ss. of serum, and there were also slight adhesions between it and the heart.

The right ventricle was enlarged to one-third more than its usual size. In it a firm coagulum, with well organised, extremely vascular points. The coagulum in the left ventricle was much softer than the right.

The left ventricle was dilated to double its natural size, and its walls in the centre were seven-twelfths of an inch in thickness. The one lip of the mitral valve was natural, the other shrunken and contracted, leaving the orifice patescent. The lining membrane of the ventricle was opaque.

The aorta at its arch was healthy, smooth and natural.

57

The right ventricle was one-third of an inch in thickness, firm, and the internal surface smooth. Valves of the pulmonary artery flexible but reddened. The right auricle was dilated.

A large coagulum is attached to the semilunar valves. From one of them arises a concretion of a reddish tint, composed partly of calcareous matter, partly of the cheesy substance often found in aortitis. This mass involves two of the valves, the structure of which is totally destroyed. It is thirteen lines in length, and at the extremity half an inch in width, and projects a third of an inch into the calibre of the tube at its base, encroaching on the third valve, which, as just mentioned, is the point of normal structure to which it is attached. It is obvious that the opening must have been extremely contracted, and hence offered a rough surface to the passage of the blood; while on the other hand the closing of the valves was entirely out of the question. The valve which was least diseased offers an ulceration upon its surface with inverted edges, and is semi-cartilaginous and much reddened.

[*Remarks.*—There are many points of much interest connected with the case, which would have been still more numerous if the notes had been more full.

At the time of the entrance of the patient he was obviously labouring under advanced disease of the kidneys; his urine was highly albuminous, and the complexion was of the peculiar tint usually found in renal dropsy. The patient was dull of intelligence, and spoke only a half intelligible German patois, so that it was impossible to obtain even a tolerable history of the case; and there were no means of knowing whether the disease of the heart or kidneys had first occurred. The cardiac affection, at the time of his entrance, was apparently limited to the patescence of the mitral valve, and roughening and thickening of the semilunar. The ulceration and morbid concretion were more recent changes, and I have no doubt originated with the attack of fever, which was referred to aortitis, partly from the character of the pulse, but much more from the great increase in the morbid sounds of the heart, especially the second sound, which was rendered extremely harsh, from the regurgitation of the blood at the orifice of the aorta, and its meeting with the rough and projecting mass.

Not the least interesting fact in the case, was the latent arachnitis. This is not very unusual in cases of extensive lesion of the kidneys; the functional disorder, which is the usual termination of such cases, gradually passes into inflammation. The inflammation at the orifice of the aorta was probably owing to the same cause—the cachexia produced by the renal disease. Dr. Chevers, in a recent number of Guy's Hospital reports, lays great stress upon this form of aortitis, and the peculiar condition of the body in which it occurs. The termination of inflammation of this character is almost necessarily fatal, and is, of course, beyond the reach of the ordinary modes of treatment. Erysipelas accompanied the earliest symptoms of the inflammation, confirming the analogy pointed out by Dr. Chevers, between erysipelas and the asthenic forms of aortitis.—W. W. G.]

